# Genomes and Transcriptomes of Duckweeds

**DOI:** 10.3389/fchem.2018.00230

**Published:** 2018-06-20

**Authors:** Dong An, Changsheng Li, Yong Zhou, Yongrui Wu, Wenqin Wang

**Affiliations:** ^1^Department of Plant Sciences, School of Agriculture and Biology, Shanghai Jiao Tong University, Shanghai, China; ^2^National Key Laboratory of Plant Molecular Genetics, CAS Center for Excellence in Molecular Plant Sciences, Shanghai Institute of Plant Physiology and Ecology, Chinese Academy of Sciences, Shanghai, China

**Keywords:** duckweeds, genome size, genome, transcriptome, gene family

## Abstract

Duckweeds (*Lemnaceae* family) are the smallest flowering plants that adapt to the aquatic environment. They are regarded as the promising sustainable feedstock with the characteristics of high starch storage, fast propagation, and global distribution. The duckweed genome size varies 13-fold ranging from 150 Mb in *Spirodela polyrhiza* to 1,881 Mb in *Wolffia arrhiza*. With the development of sequencing technology and bioinformatics, five duckweed genomes from *Spirodela* and *Lemna* genera are sequenced and assembled. The genome annotations discover that they share similar protein orthologs, whereas the repeat contents could mainly explain the genome size difference. The gene families responsible for cell growth and expansion, lignin biosynthesis, and flowering are greatly contracted. However, the gene family of glutamate synthase has experienced expansion, indicating their significance in ammonia assimilation and nitrogen transport. The transcriptome is comprehensively sequenced for the genera of *Spirodela, Landoltia*, and *Lemna*, including various treatments such as abscisic acid, radiation, heavy metal, and starvation. The analysis of the underlying molecular mechanism and the regulatory network would accelerate their applications in the fields of bioenergy and phytoremediation. The comparative genomics has shown that duckweed genomes contain relatively low gene numbers and more contracted gene families, which may be in parallel with their highly reduced morphology with a simple leaf and primary roots. Still, we are waiting for the advancement of the long read sequencing technology to resolve the complex genomes and transcriptomes for unsequenced *Wolffiella* and *Wolffia* due to the large genome sizes and the similarity in their polyploidy.

## Introduction

The *Lemnaceae* family, commonly known as duckweeds, comprises five genera of *Spirodela, Landoltia, Lemna, Wolffiella*, and *Wolffia* within the monocot order of Alismatales. Each duckweed species presents their unique features. The *Spirodela* species are served as a promising aquatic reference genome due to its small genome size. The *Landoltia* and *Lemna* species are explored for applications in phytoremediation especitally in waste water treatment. The *Wolffiella*, and *Wolffia* are becoming renewable biorefinery feedstock given their fast growth and high starch content (Cheng and Stomp, 2009). Most duckweeds reproduce next generations by vegetative budding during spring and summer, while they become natural starch repository when they switch into a dormant stage during winter time (Landolt, 1986). They are tolerant to various stresses, such as heavy metal, irradiations, and ${\text{NH}}_{4}^{+}$, serving an ideal system to study the response to abiotic stresses and providing an efficient way to restore the environment (Wang W. et al., 2016; Van Hoeck et al., 2017; Xu et al., 2018). Duckweeds can be co-cultured with municipal or swine wastewater to remove excess nitrogen and phosphorus, while the biomass can be readily converted into ethanol (Cheng and Stomp, 2009). Their unique characteristics and the economic potential have attracted a broad interest including (i) fast biomass accumulation, (ii) no competition with arable land, (iii) phytoremediation of wastewater or heavy metal polluted water, (iv) biofactories for pharmaceutical drugs (Stomp and El-Gewely, 2005), and (v) high starch content. To further release and improve the potential capabilities, it is becoming critical to interpreting the genome sequence, structure and gene functions. The falling cost of next generation sequencing (NGS) has made it accessible to an individual laboratory (Alkan et al., 2011). Still, sequencing a genome is a non-trivial work. It remains a challenging task within the reach of non-experts in terms of obtaining a high-quality assembly and annotation. The NGS reads are too short to resolve the high repetitive elements and polyploidy known in plant genomes, leading to incomplete or ambiguous assemblies (Li et al., 2018). Thus, the choice of plant genomes for sequencing has been driven mainly by cost efficiency and the avoidance of complexity. Thus, the species of *Spirodela* and *Lemna* with smaller genome size and less complexity have undergone genome sequencing (Wang et al., 2014a; Van Hoeck et al., 2015). The larger genomes of *Wolffiella* and *Wolffia* are not sequenced yet.

**Figure 1:**
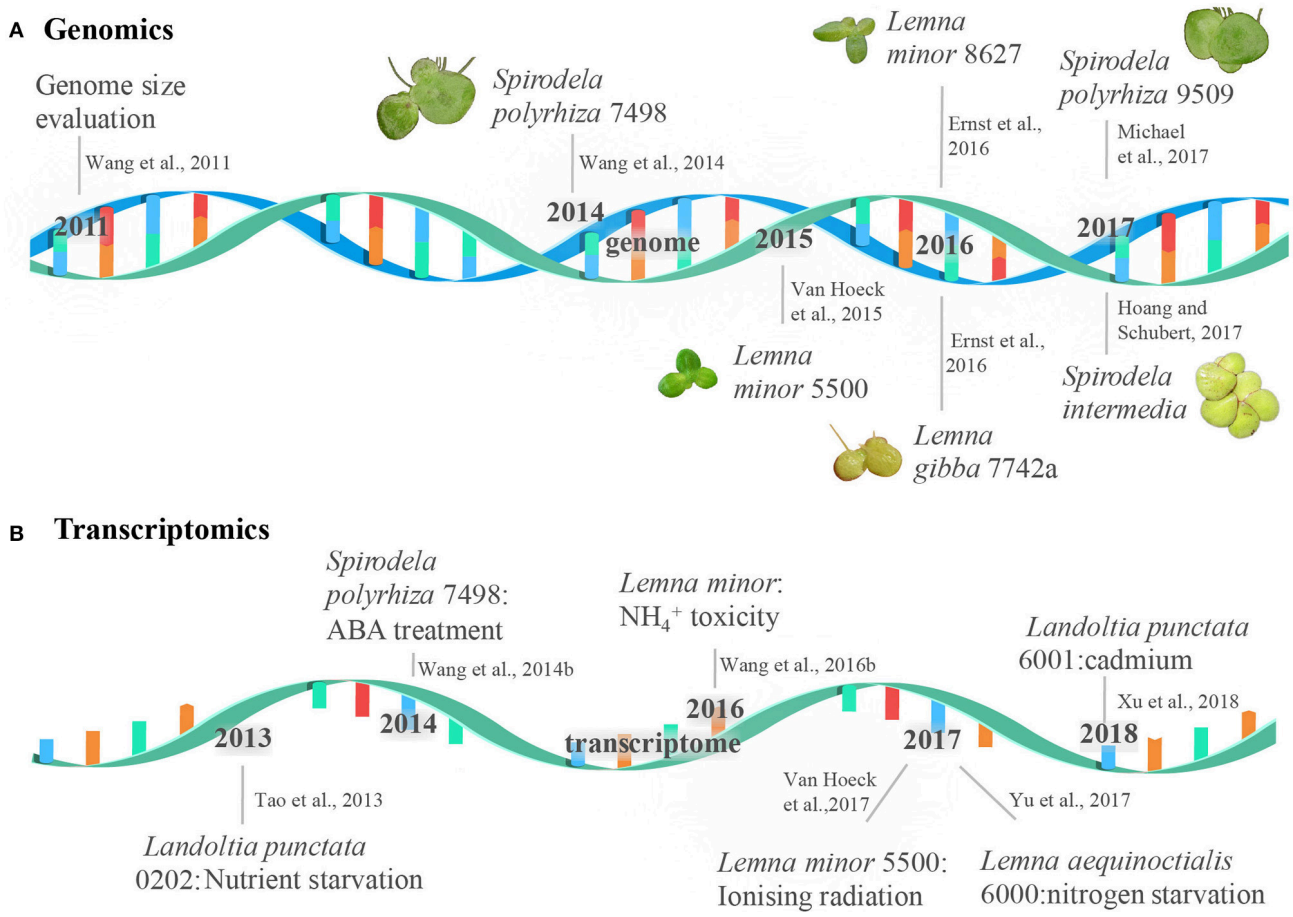
The milestone of duckweed genome and transcriptome sequencing. The events of the genome and transcriptome sequencing were shown in a double-stranded DNA molecule **(A)** and a single-stranded RNA molecule **(B)**, respectively.

The alternative way is to only sequence RNA, the transcribed region from DNA template, which is the most efficient approach to get the essential information from the genome (Roberts et al., 2011; Trapnell et al., 2012). Transcriptome analysis of duckweeds is a great resource to understand how duckweeds get adapted to aquatic environments and seasonal changes, and how they respond to abiotic stresses. The invaluable resource will set the framework and stimulate new insights into discovering duckweeds' potential. In this review, we primarily introduce the sequencing strategy, the feature of genome and transcriptome along with the unique biology and physiology for the genera of *Spirodela, Landoltia*, and *Lemna*. The milestone of duckweed genome and transcriptome sequencing is summarized in Figure 1. We also give readers the future perspectives on sequencing the complex genome and transcriptome of duckweeds by using long read sequencing and the applications of the sequenced genomes in duckweeds research.

## Genome sizing

With the fast development of sequencing technology especially NGS, the falling cost makes genome sequencing accessible to an individual laboratory. Still, genome assembly is a challenging task with respect to the inherent repeat content in DNA, the read length, and the sequencing depth. The cost-effective way is to choose the smallest genome that contains fewer repeats and is sequenced with a deeper coverage. The comprehensive study of genome sizing 23 duckweed species across 115 accessions were conducted by flow cytometry (FCM) before the initiation of duckweed genome sequencing. The ranges of genome size for each genus were summarized, presenting the big picture of genome variation in the duckweed family. It was found that the duckweed genome sizes varied 13-fold, ranging from 150 Mb in *Spirodela polyrhiza* to 1,881 Mb in *Wolffia arrhiza*. The 158-Mb genome of *Spirodela polyrhiza* 7498 was selected for sequencing. Surprisingly, the genome sizes of duckweeds correlated negatively with plant leaf (frond) size. The genus of *Landoltia* had a relatively stable genome size of ~380 Mb, while *Lemna* showed a significant intraspecific and interspecific variation from 323 to 760 Mb, indicating polyploidy might be a major mechanism for the genome change (Soltis et al., 2015; Segraves, 2017; Van de Peer et al., 2017). The *Wolffiella* and *Wolffia* had the biggest genomes of 973 and 1,881 Mb, respectively (Wang et al., 2011). Given the small genome sizes of *Spirodela* (Wang et al., 2014a; Bog et al., 2015; Michael et al., 2017) and *Lemna* (Van Hoeck et al., 2015), they were selected for the first round of genome sequencing. The broad range of genome sizes makes duckweeds an invaluable system to study polyploidization and genome evolution.

The chromosome numbers reported for duckweeds were from *2n* = 20 to 126 with high variability (Landolt, 1986). Compared with Arabidopsis (5 chromosomes with a genome size of 157 Mb) (Bennett et al., 2003) and rice (12 chromosomes with a genome size of 466 Mb) (Yu et al., 2002), the individual chromosome size of *Spirodela polyrhiza* is small due to 20 chromosomes with 158 Mb, whereas it was reported that there was no obvious correlation between chromosome number and genome size (Hoang and Schubert, 2017).

The epigenetic modifications in duckweed chromatin played a significant role in gene transcription and translation. The studies of histone methylation and DNA modification including heterochromatic 5-mC, H3K9me2 and H3K27me1 in interphase nuclei found that the duckweeds with the genome size range of 158 to 1,881 Mb showed dispersed distribution of heterochromatin signatures. The immunolabelling pattern was similar to the early developmental stages of Arabidopsis nuclei, implying the association with the rapid growth of duckweeds but less dependent on the DNA content (Cao et al., 2015).

## *Spirodela* genome and transcriptome

The morphology of *Spirodela* is very simple with only leaves and roots. The genus has two species of *Spirodela polyrhiza* and *Spirodela intermedia* but multiple ecotypes (Les et al., 2002; Bog et al., 2015). The genome sequencing project indicated that it was subjected two ancient rounds of Alismatales-specific whole genome duplications (WGDs) and likely eliminated non-essential protein-coding genes, rDNA, and repeat elements to maintain its small genome size (Michael et al., 2017).

### Genomes of *spirodela polyrhiza*

With the confirmation of the smallest genome of 158 Mb in the duckweed family and its basal ancestral phylogenetic position among duckweeds, *Spirodela polyrhiza* 7498 (Sp7498) was selected to whole genome sequencing as a basal monocot reference (Wang et al., 2011). With the prosperous development of the high-throughput DNA sequencing technology, the platform of 454 Life Sciences based on the “sequencing by synthesis” principle was used to sequence the genome of Sp7498, producing 21X coverage with 400–500 bp read lengths. BAC-end sequencing (BACs: Bacterial Artificial Chromosomes) from 15,260 clones with 100 Kb insertions was conducted by Sanger technology (Table 1). A complete physical map was developed by fingerprinting the BAC library with 10X coverage, providing an essential framework to order and join contigs assembled from 454 reads and BAC-end sequences (Wang et al., 2014a). To investigate the genome-wide intraspecific variation in *Spirodela* populations, another ecotype of *Spirodela polyrhiza* 9509 (Sp9509) was recently sequenced with 95X Illumina short reads and high-throughput genome mapping technology (Michael et al., 2017).

**Table 1:** The parameters for sequencing, assembling and annotating duckweed genomes.

**Species**	**Genome size (Mb)**	**Platform**	**Sequencing coverage**	**Assembly program**	**Scaffold #**	**Scaffold N50**	**Contig N50 (Kb)**	**Repeat (%)**	**Protein coding gene #**	**References**
*Spirodela polyrhiza* 7498	158	454 and Sanger	21	Newbler	32	3.8 Mb	18	17	19,623	Wang et al., 2014a
*Spirodela polyrhiza* 9509	160	Illumina and BioNano	95	AllPathsLG and SSPACE	20	7.6 Mb	19	23.8	18,507	Michael et al., 2017
*Lemna minor* 5500	481	Illumina	120	SOAPdenovo2 and SSPACE	46,105	23.6 Kb	20.9	61.5	22,382	Van Hoeck et al., 2015
*Lemna minor* 8627	800	Illumina and PacBio	NA	NA	NA	NA	222	NA	NA	Ernst, 2016
*Lemna gibba* 7742a	450	Illumina	NA	NA	NA	520 Kb	53	NA	21,830	Ernst, 2016

The final genome assembly for Sp7498 obtained 32 pseudomolecules with a contig N50 of 18 Kb and an N50 scaffold of 3.8 Mb, leaving about 10% unresolved gaps that could be repeat elements which were challenging to be assembled due to the limitation of the short reads (Table 1). By multicolor fluorescence *in situ* hybridization (mcFISH) using 96 BACs as probes with little repetitive sequences, the originally assembled 32 pseudomolecules were assigned into 20 chromosomes with an average resolution of 0.89 Mb (Wang et al., 2014a). The genome of Sp9509 was *de novo* assembled into 774 scaffolds with a contig N50 of 19 kb and a scaffold N50 of 4.3 Mb. With the guide of the previous assembled Sp7498 genome, Sp9509 scaffolds were joined into 23 larger ones with an N50 length of 5.8 Mb. To further close gaps and validate the assembled accuracy, a genome-wide physical map using the BioNano Genomics Iris System was developed for Sp9509 genome, which was resolved into 20 chromosomes with a scaffold N50 of 7.6 Mb (Table 1). It was found that the 20 chromosomes of *Spirodela* could be originated from seven ancestral chromosome blocks with two rounds of WGDs 95 million years (Myr) ago (Cao et al., 2016). The chromosomally integrated genome has accelerated the study of karyotype evolution in duckweed species.

The genome alignment between Sp7498 and Sp9509 revealed conflicts and identified potential misassembled sites in each genome, indicating that more PCR validations or long reads spanning over the junctions were required. There were 96 high-confidence structure variations (SVs) with the range of 1,000 to 100,000 bp between the two BioNano genome maps. The estimation of 81 rDNA copies was found in Sp9509, extremely less than Arabidopsis (570) and maize (12,000). The Southern blot analysis of four different accessions of *S. polyrhiza* revealed that the copy number of the rDNA clusters was all <100 copies (Michael et al., 2017).

The identified repeat elements constituted of ~17% of the Sp7498 genome, most of which were long terminal repeat (LTR)-retrotransposons (Wang et al., 2014a). There were 25.25% repeats with 271 full-length long terminal repeats (LTRs). Comparative analysis with other species of Brachypodium, rice, and sorghum, *Spirodela* showed low transposon similarity, indicating a large evolutionary distance between *Spirodela* and the other monocots. The study of genome-wide bisulfite-sequencing found that the extent of cytosine methylation was higher for transposable elements than gene regions. The overall DNA methylation level in Spirodela was estimated to be 9%, which was considered to be the lowest in the tested plants of *A. thaliana* (32%), rice (39%), *Setaria italica* (44%) and *B. distachyon* (54%) (Michael et al., 2017).

Generally the high copy number tandem repeats (TRs) exist in the centromeres of a genome (Melters et al., 2013). The Sp7498 genome was predicted to have a 138-bp centromere repeat-like sequence, whereas Sp9509 was found a 119-bp TR on 19 out of the 20 chromosomes that contained high DNA methylation levels (Michael et al., 2017). The distribution of the 119-bp centromere repeat across some of the Spirodela chromosomes suggested that they were holocentric. This result was consistent with a dispersed heterochromatin signal observed in cytological studies (Cao et al., 2015).

The bioinformatics analysis predicted that there were 59 conserved microRNAs (miRNAs) of 22 families and 25 novel miRNAs. The small RNA sequencing validated 29 *Spirodela*-specific miRNAs in the genome of Sp9509. The sequence-based annotation identified five and three loci for miRNA156 and miRNA159 in Sp9509, respectively (Michael et al., 2017). In contrast, the Sp7498 genome included 24 loci encoded for miRNA156 and one locus encoded for miRNA159 (Wang et al., 2014a).

*Spirodela polyrhiza* has a small genome that is similar to Arabidopsis, but it has 30% fewer protein-coding genes of 19,623 in Sp7498 and 18,507 in Sp9509. Although the *Spirodela* genome exhibited reduced gene content, it shared a number of 8,255 common gene families with other plant species of Arabidopsis, tomato, banana, and rice (Wang et al., 2014a). Examination of copy number variation in *Spirodela* could give us indications of its compact and reduced morphogenesis, aquatic suspension and suppression of juvenile-to-adult transition.

Lignin is a major component of secondary cell walls to support land plants' up-straight height. It was found that duckweeds contained very little lignin and cellulose content possibly owing to their specific aquatic habitat (Blazey and Mcclure, 1968). There were 141 and 156 gene copy numbers for lignin biosynthesis in sorghum and rice, respectively, while the *Spirodela* had only 70 members, consistent with its floating habitat that requires little strength to hold up their weight (Table 2; Wang et al., 2014a). Cellulose biosynthesis is critical for all plant cell wall synthesis. The gene families of cellulose synthase-like genes (CSL) and glycosyl transferases (GT31) for cell wall biogenesis were contracted in *Spirodela* in comparison with Arabidopsis and rice (Table 2) that was consistent with the low cellulose content in *Spirodela* (Wang et al., 2014a). Consistent with Sp7498, the gene family of expansin in Sp9509 was reduced to 14 in comparison of 36 members from Arabidopsis.

**Table 2:** The contracted, expanded and conserved gene families in *Spirodela* compared with Arabidopsis and rice.

	**Contracted gene family**				**Expanded gene family**		**Conserved gene family**			
**Species**	Lignin biosynthesis^*^	**Cellulose biosynthesis gene**		**Expansin**	**Glutamate synthase genes**	**miRNA156**	**Starch biosynthesis genes**			
		**CSL**	**GT31**				**AGPase**	**Starch synthase**	**Starch branching**	**Starch debranching**
Spirodela	70	21	21	14	11	32	4	5	3	4
Arabidopsis	63	29	33	36	2	10	6	5	2	4
rice	156	36	39	-	3	19	7	10	3	4

* *Lignin biosynthesis involves 10 gene families. Here shows the total copy number of 10 gene families. CSL, Cellulose synthase-like genes; GT, Glycosyl transferase; AGPase, ADP-glucose pyrophosphorylase*.

The fast growth of duckweeds needs highly efficient absorption of nutrients, for example, glutamate synthase genes that are dedicated to ammonia assimilation for duckweed fast propagation. It was found that up to four-time copies of glutamate synthase genes in *Spirodela* compared to Arabidopsis and rice (Table 2; Wang et al., 2014a; Michael et al., 2017). Consistent with rare flowering phenotype, miRNA156 playing a negative role in flowering and suppressing the juvenile-to-adult transition was identified to increase its copy numbers in *Spirodela* (Table 2). However, the copy numbers of starch biosynthesis genes encoding for ADP-glucose pyrophosphorylase (AGPase), starch synthase, starch branching enzyme, and starch debranching enzyme remained constant in *Spirodela*, Arabidopsis and rice (Table 2), indicating their conserved functions in starch biosynthesis.

Another aquatic plant *Zostera marina*, which is closely relative to *S. polyrhiza*, contains a genome size of 202.3 Mb and encodes 20,450 protein-coding genes, which is comparable with Sp7498. However, compared to 17% repeat elements in Sp7498, repeat elements in *Z. marina* accounted for 63% of the assembled genome. The gene family analysis showed that both species had gained 600 but lost 2000 gene families. The gene families of terpenoid genes, carbohydrate sulfotransferases, sulfatases, and MADS-box transcription factors responsible for flowering were dramatically reduced, indicating structural and physiological adaptations to their lifestyles and consistent with previous results (Olsen et al., 2016).

### Chromosome reconstruction of *spirodela intermedia*

The species of *Spirodela intermedia* has a similar genome size with *Spirodela polyrhiza*, while the genome has not been sequenced. The homology and chromosome rearrangements were investigated in *Spirodela intermedia* compared with the reference of *Spirodela polyrhiza* by using mcFISH with the identical set of 96 BACs from 20 chromosome pairs (Cao et al., 2016; Hoang and Schubert, 2017). It was found that *S. intermedia* was reconstructed into 18 chromosome pairs, two less than *S. polyrhiza*. Ten chromosome pairs were proved to be conserved between the two species, while six new linkages were detected possibly due to the rearrangements of chromosome breakage and translocations in *S. intermedia*. The reconstruction of karyotype provides a basis to study chromosome evolution in the genus of *Spirodela* and to assist *S. intermedia* genome assembly in future.

### Transcriptome of *spirodela polyrhiza* treated with abscisic acid

The genome sequence of *S. polyrhiza* provides a reference to analyse a whole transcriptome shotgun sequencing, also called RNA sequencing (RNA-Seq). RNA-Seq is a common technique to quantify gene expressions during plant development or stress stimuli. It was known that *S. polyrhiza* could survive through the cold winter or other extreme stress conditions by producing dormant fronds (turions) that were abundant of starch content. The addition of the hormone of abscisic acid (ABA) into growth medium can lead to turion formation quickly and starch synthesis (Smart and Trewavas, 1983). To better understand the mechanism of starch accumulation, RNA-Seq was conducted for the developing turions treated with ABA and the differentially expressed genes (DEGs) were defined. The functional terms of seed dehydration, carbohydrate, secondary metabolism, and senescence were enriched in up-regulated DEGs, whereas the genes responsible for rapid growth and biomass accumulation and protein synthesis were down-regulated (Table 3; Wang et al., 2014b). The identification and functional annotation of DEGs set a framework to understand the regulation of starch synthesis and the mechanism of dormancy. Moreover, the candidate genes could be further validated and engineered for practical applications such as starch and ethanol production.

**Table 3:** The summary of RNA-Seq studies in duckweeds.

**Species**	**Treatment**	**Assembled contig ^#^**	**Assembled contig N50**	**Enriched biological processes**		**SRA**	**Platform**	**References**
				**Up-regulated**	**Down-regulated**			
*Spirodela polyrhiza*	ABA induced turion formation	NA	NA	Seed dehydration, carbohydrate and secondary metabolism, and senescence	Rapid growth and biomass accumulation, carbon fixation, and protein synthesis	PRJNA205940	ABI_SOLID	Wang et al., 2014b
*Landoltia punctata*	nutrient starvation^#^	74,797	1,928	Starch accumulation, flavonoid biosynthesis, some ion transporters	Photosynthesis and respiration	PRJNA185389	Illumina HiSeq2000	Tao et al., 2013
	nutrient starvation^#^	155,903	2,190	Flavonoid and anthocyanin biosynthesis	Lignin biosynthesis	PRJNA185389	Illumina HiSeq2000	Tao et al., 2017
	cadmium toxicity	NA	NA	DNA repair, ROS metabolism, vacuolar sequestration, and protein metabolism	Protein phosphorylation, cellulose biosynthetic process, and cell wall organization	PRJNA361433	Illumina HiSeq4000	Xu et al., 2018
*Lemna minor*	ionizing radiation^#^	NA	NA	Cell wall modification, floavonoid biosynthesis, oxidative stress	DNA repair and mitosis	NA	Illumina HiSeq2000	Van Hoeck et al., 2017
	${\text{NH}}_{4}^{+}$ toxicity	71,094	988	ROS scavenging, programmed cell death (PCD), and ligin biosynthesis	Ribosome pathway	PRJNA302233	Illumina HiSeq2500	Wang W. et al., 2016
*Lemna aequinoctialis* 6000	nitrogen starvation^#^	72,105	1,233	Starch biosynthesis	Nitrate reductase, glutamine synthase, and glutamate synthase	PRJNA368628	Illumina HiSeq2000	Yu et al., 2017

# *Nutrient starvation means that the duckweeds were transferred from nutrient-rich solution to distilled water. Nitrogen starvation was the treatment of duckweeds without any nitrogen in the medium. Ionizing radiation includes gamma- and beta-radiation with the addition of ^137^Cs and a ^90^Sr source in the nutrient medium*.

## *Landoltia* transcriptome

*Landoltia* genus contains only one species named *Landoltia punctata*. It can be readily distinguished from other duckweed species by root number in spite that is used to be referred as *Spirodela oligorrhiza*. *Landoltia* has a typical number of 2–4 roots for each frond in comparison with one root for *Lemna* and more than five roots for *Spirodela*.

### Transcriptome of *landoltia punctata* treated with nutrient starvation

It was reported that *L. punctata* contained high starch, rich flavonoid but little lignin during nutrient starvation, showing their potential to be developed as a resource plant for biofuel fermentation and flavonoid extraction. The starch percentage in *L. punctata* treated with nutrient starvation can reach 45.4% of the dry weight. The activity of ADP-glucose pyrophosphorylase, the most important key enzyme involved in starch synthesis was increased from the initial 9.6 units to 14.7 units per mg of total protein (Tao et al., 2013). A comprehensive transcriptome study (RNA-Seq) was conducted by transferring *L. punctata* to distilled water lack of nutrients (Table 3). Without *Landoltia* genome reference, short RNA-Seq reads were *de novo* assembled in order to build the transcriptome reference. A number of 74,797 contigs more than 200 bp were obtained. The N50 length of these contigs was 1,928 bp and the maximum length was 16,562 bp. The BLASTX found that 51,968 had significant hits that matched 25,581 unique protein accessions (Tao et al., 2013).

The short reads were aligned back to the assembled 74,797 contigs to quantify the gene expression profiling under nutrient starvation. The results showed that the transcripts encoding for key enzymes involved in starch and flavonoid biosynthesis were up-regulated, while the transcripts for photosynthesis and the rate-limiting enzymes of lignification were down-regulated (Tao et al., 2013).

A further investigation focused on flavonoid content was performed growing *L. punctata* in different culture medium (Tao et al., 2017; Table 3). Metabolome analysis detected a flavonoid accumulation from the original 4.51 to 5.56% (dry weight) after growing in distilled water. Consistent with metabolome, transcriptome analyses proposed that a special phenylalanine metabolic flux lead to the high flavonoid but the low lignin content. The integration of transcriptome, proteome, and metabolome indicated that high biomass with low starch and stable flavonoid content was produced in the full nutrient medium, while the accumulation of starch and flavonoids were stimulated by nutrient starvation. The plant growth retardant of uniconazole inhibited flavonoid biosynthesis but increase starch accumulation (Tao et al., 2017).

### Transcriptome *landoltia punctata* treated with the heavy metal of cadmium

*Landoltia punctata* is an efficient, green, and economic approach to remove heavy metals and other pollutions from the water. Cadmium (Cd) is a heavy metal that is detrimental to the environment and crops. The screening test on 200 duckweed clones showed that *L. punctata* 6001 exhibited Cd tolerance. To further explore the molecular mechanism underlying the resistance to the heavy metal, a high-throughput transcriptome analysis was carried out for the Cd-treated samples (Table 3). DEG clustering and enrichment analysis showed the biological processes of DNA repair, ROS metabolism, vascuolar sequestration, and protein metabolism played a crucial role in Cd response and detoxification. Furthermore, the carbohydrate metabolic flux tended to be modulated in response to Cd stress (Xu et al., 2018).

The transcriptome sequence could not give the complete picture of the whole genome in terms of intergenic regions, introns, and repeats that could not be transcribed. Still, it provides a comprehensive view of gene expression with enough sensitivity and accuracy at certain developmental stages and under specific conditions. So far, none of *Landoltia punctata* has been sequenced. Therefore, the transcriptome sequence is particularly invaluable to understand their response to abiotic stresses and to benefit the basic research and practical applications.

## *Lemna* genome and transcriptome

*Lemna* is a duckweed genus that is adapted to a broad climate region and extensively used in labs. The species of *Lemna minor* and *Lemna gibba* are the model systems to understand fundamental plant research such as the circadian clock, flowering mechanism, and genetic transformation (Yamamoto et al., 2000; Cedergreen and Madsen, 2002; Miwa et al., 2006). *Lemna gibba* is an aquatic higher plant that is used to evaluate the toxicity of pesticides by the Environmental Protection Agency (EPA) due to the facts of direct assimilation of chemicals from a liquid medium and their rapid growth (Brain and Solomon, 2007).

### *Lemna minor* 5500 genome

Given the invaluable potential of *L. minor* for the physiology research and biotechnological applications, the accession of 5500 (Lm5500) with 481 Mb genome was assembled and annotated (Van Hoeck et al., 2015). The paired-end sequencing of HiSeq library covered 90X of the genome, while the MiSeq library represented 30X. The bioinformatic pipeline generated a moderate assembly including 49,027 contigs (N50 contig size 20.9 Kb) and 46,105 scaffolds (N50 scaffold size 23.6 Kb). It was well-known that the short reads could hardly span long repetitive sequences in a typical plant genome. The lack of mate-pair libraries, fosmids or BAC clones with large insertions had led to higher reduced contiguity for Lm5500 than Sp7498 (Xu et al., 2018). It was revealed that the Lm5500 genome contained 62% repetitive sequences including 31.20% retrotransposons, 5.08% DNA transposons, 3.91% tandem repeats, and 21.27% of other unclassified repeats (Van Hoeck et al., 2015). In comparison with ~17% repeats in 158 Mb *S. polyrhiza*, repetitive elements in 481 Mb *L. minor* could explain 94.5% of the genome size difference. The structural annotation showed the average gene length was 2,738 bp comprising of 1,332 bp CDS, 208 bp exon, and 209 bp intron. The mean exon number per gene was 4.8. A number of 22,382 protein-coding genes were predicted in *L. minor*, similar to 19,623 members of *S. polyrhiza* (Table 1; Wang et al., 2014a). There were 66.2% of *Lemna* proteome shared with the *Spirodela*. The GO analysis revealed that the gene functions involved in environmental adaptation, biomass production, and response to abiotic stresses were enriched. For example, the genes encoded for glutamine synthetases (GSs) and glutamate synthases (GOGATs) were greatly expanded to 12 and 21 members in *L. minor*, compared with 7 and 11 ones in *S. polyrhiza*, respectively, indicating their potential in wastewater remediation and fast growth.

### *Lemna minor* 8627 genome

Another genome project of 800 Mb *Lemna minor* 8627 (Lm8627) was done by Martienssen Lab. *Lemna minor* had the ability to remove nitrogen and phosphorus from swine lagoon water (Stomp and El-Gewely, 2005). It was also an efficient system for genetic transformation using agrobacterium-mediated gene transfer protocol (Yamamoto et al., 2000; Cedergreen and Madsen, 2002). Lm8627 had nearly two times of Lm5500 genome size (481 Mb) and more than five times of Sp7498 (158 Mb). It was proposed that the *Lemna* ancestor experienced at least one recent WGD after the split of *Lemna* and *Spirodela*. The different degree of gene removal from duplicated genome resulted in various *Lemna* species. It was also hypothesized that the mechanisms of large repeat expansion or very recent WGD could be possible. The more sequenced genomes will clarify the relationships and the evolution history (Van Hoeck et al., 2015). The draft genome of *L. minor* 8627 was highly fragmented with a contig N50 of 65 Kb over nearly 40,000 contigs. The addition of long reads from PacBio sequencing greatly improved the contig N50 to 222 Kb and highly reduced the contig number to fewer than 9,000 (Table 1; Appenroth et al., 2015; Ernst, 2016). The genomic data can be retrieved from the database (https://www.Lemna.org), while the annotation is in progress.

### *Lemna gibba* 7742a genome

To expedite the biology and genetics study for aquatic plants, Evan Ernst from Martienssen Lab sequenced 450 Mb L. gibba 7742a (Lg7742a) genome by Illumina short reads. The preliminary assembly of Lg7742a displayed fragmentation due to the absence of a physical or genetic map with a contig N50 of 53 Kb and a scaffold N50 of 520 Kb. The most recent annotation found 21,830 protein-coding genes (Table 1; Ernst, 2016), close to Lg5500 and Sp7498. The peer-reviewed website (https://www.Lemna.org) created by Cold Spring Harbor Laboratory (CSHL) made the draft genome sequence of *Lemna gibba* accessible to the community before their publication. The analysis tools of Gbrowse and BLAST were also available online.

The present version of *Lemna* genome will be highly helpful for deciphering the functional genomics contributed to absorbing nutrition from waste water and boosting the biomass accumulation.

### Transcriptome of *lemna minor* treated with radiations and ${\text{NH}}_{4}^{+}$

Plants are constantly exposed to various radiations. The injury of chlorophyll and the depletion starch synthesis were observed under radiation (Farooq et al., 2000). To provide a better understanding of environmental radiation exposure, the gene expression responses measured by RNA-Seq were evaluated under different dose of gamma- and beta-radiation for 7 days (Table 3). The functional analysis revealed that *L. minor* could tolerate the lower dose of radiation by triggering the cell wall modification and flavonoid biosynthesis. However, the gene expression involved in anti-oxidative defense systems and ATP production were up-regulated, while the regulations of DNA repair and mitosis were decreased at the high dose of radiation (Van Hoeck et al., 2017).

Different with other plants, *L. minor* could grow well even in the high ${\text{NH}}_{4}^{+}$ environment, whereas little knowledge of the tolerance mechanism was known. Thus, the comparative transcriptome of *L. minor* under high concentration of ${\text{NH}}_{4}^{+}$ was studied (Table 3; Wang W. et al., 2016). It was reported that the genes encoding ROS scavenging enzymes, such as superoxide dismutase and peroxidase were detected to be up-regulated by ${\text{NH}}_{4}^{+}$ treatment. The increased lignin biosynthesis might also contribute to the resistance of ${\text{NH}}_{4}^{+}$ toxicity.

### Transcriptome of *lemna aequinoctialis* treated with nitrogen starvation

*Lemna aequinoctialis* 6,000 was sampled from Hunan Province of China. It was found that the starch content after 9 days of nitrogen starvation was three times higher compared to the pre-treatment. To identify the genes responsible for starch accumulation, the transcriptome profile of *L. aequinoctialis* 6000 was examined using RNA-Seq (Table 3). The generated sequencing data (25 Gb) were *de novo* assembled into 72,105 unigenes with an average length of 1,233 bp. The genes involved in nitrogen metabolism exhibited the earliest responses to nitrogen stress, whereas the genes responsible for carbohydrate biosynthesis were regulated subsequently. The expression of genes encoding nitrate reductase, glutamine synthetase, and glutamate synthase were down-regulated under nitrogen starvation. Consistent with the change of starch content, the activity of AGPase was significantly increased. It was concluded that the increase of ADP-glucose and starch contents under nitrogen starvation was a consequence of increased output from the gluconeogenesis and TCA pathways (Yu et al., 2017). The identified genes would be promising candidates for further genetic improvement of starch production. The mechanisms of starch accumulation during nitrogen starvation provided a foundation for the improvement of bioethanol production (Yu et al., 2017).

## Organellar genomes

The chloroplast genomes of *Spirodela polyrhiza* (Sp7498), *Wolffiella lingulata* (Wl7289), and *Wolffia Australiana* (Wa7733) were assembled by computationally selection filter with a copy number-sensitive algorithm from total DNA sequencing (Wang and Messing, 2011). The cpDNA was a circular molecule of 168,704–169,353 bp containing a pair of 31,683–31,930 bp inverted repeat regions (IRs). Comparative analysis suggested that the chloroplast genome was conserved in gene number and organization with respect to the reference genome of *L. minor* (Mardanov et al., 2008). However, substantial variations including nucleotide substitution, deletions and insertions occurred in non-coding regions compared to the chloroplast genomes of grass family (Wang and Messing, 2011).

The first complete mitochondrial genome of duckweeds was *Spirodela polyrhiza* assembled from ~100x coverage of raw reads. The 228,493-bp genome was annotated into 57 genes (35 protein-coding genes, 3 ribosomal RNAs, and 19 tRNAs) (Logan, 2006; Wang et al., 2012). Further sequence analysis showed that 4.1% of the mtDNA was originated from chloroplast DNA, but very few nuclear sequences were found in mitochondrial DNA. The phylogenetic tree suggested that Spirodela shared a common ancestor with other monocots, but there is no obvious synteny in mitochondrial genomes between Spirodela and rice (Wang et al., 2012).

## Future perspective

NGS facilitates the unprecedented development of duckweed genomics and transcriptomics (Tables 1, 3). With the overview of all sequenced duckweed genomes, we found that genomes of *Spirodela polyrhiza* (Sp7498 and Sp9509 with ~158 Mb), *Lemna minor* (Lm 5500 with 481 Mb and Lm 8627 with 800 Mb) and *Lemna gibba* (Lg 7742a with 450 Mb) have been assembled into the contig N50 of 18–222 Kb. The protein coding gene numbers were comparable, whereas the repeat contents increased with the genome sizes (Table 1). The other genomes for *Landoltia, Wolffiella* and *Wolffia* are not available yet due to the higher repeat content and larger genome size. Given the average length of 8 Kb of retrotransposons (Kumar and Bennetzen, 1999), the short reads from NGS cannot fully span over the repetitive regions, leading to fragmented assemblies (Phillippy, 2017). A superior way to resolve transposon repeats is to generate long reads that are enough to exceed transposon regions (Li et al., 2018). The third generation sequencing platforms including PacBio single-molecule real-time (SMRT) sequencing and Oxford Nanopore sequencing produce up to 20 and 200 Kb reads that are able to efficiently assist the complex genome assembly. For instances, the incorporation of additional PacBio single-molecule long reads with short reads significantly improved *L. minor* 8627's assembly, resulting in the contig N50 increased from 65 to 222 Kb (Ernst, 2016). It was reported in the abstracts of Plant & Animal Genome Conference XXVI in 2018 that 375 Mb *Wolffia Australiana* was sequenced by PacBio long reads and BioNano Genomics optical mapping. They resolved large contigs with ribosomal DNA and identified two tandem repeats with high copy number that could be from centromeres (https://pag.confex.com/pag/xxvi/meetingapp.cgi/Paper/28517). Oxford Nanopore Technology is another competitive long read third generation sequencing technologies. Using Nanopore sequencing, the 1.2 Gb *Solanum pennellii* genome was assembled into a contig N50 of 2.5 Mb (Schmidt et al., 2017). Without *de novo* assembly of RNA-Seq reads, the long read sequencing, called isoform sequencing can obtain the full RNA molecular sequencing that could define the complete gene structure including untranslated regions of 5′ and 3′ ends, introns, and exons (Wang B. et al., 2016). It is believed that the long read sequencing technology would be applied in duckweeds in near future that not only improves the genome assembly but also enhances our understanding of the complex transcriptome.

The duckweed genomics gives us an important scientific advance that could revolutionize many aspects of molecular biology and genetics. The multiple sequenced genomes become essential resources to study the comparative genomics that are aligned to find out the similarities and differences as well as the genome structure and function. The further characterization of genomes could reveal the dynamics of gene families, the activity of transposable elements and the patterns of genome duplications. Still, the future of genomics research would target to understand the structural and functional components embedded in genomes either by mutation or the genome editing technology of CRISPR/Cas9. It is well-known that genes and their products cooperate in a complex and interconnected network. The elucidation of the genetic pathways of how they contribute to cellular and organismal phenotypes would be critical. The long goal of genomics would be the development of the transgenic duckweeds that improve the agronomic traits or extensively apply in the industry in terms of bioenergy and phytoremediation.

## Author contributions

DA and WW did the literature search and wrote the manuscript, CL drew Figure 1, DA, CL, YW, YZ, and WW gave critical suggestions and made a proofreading.

### Conflict of interest statement

The authors declare that the research was conducted in the absence of any commercial or financial relationships that could be construed as a potential conflict of interest.
